# Compressive Video Recovery Using Block Match Multi-Frame Motion Estimation Based on Single Pixel Cameras

**DOI:** 10.3390/s16030318

**Published:** 2016-03-02

**Authors:** Sheng Bi, Xiao Zeng, Xin Tang, Shujia Qin, King Wai Chiu Lai

**Affiliations:** 1 School of Computer Science & Engineering, South China University of Technology, Guangzhou 510006, China; picy@scut.edu.cn (S.B.); zengxiao1028@gmail.com (X.Z.); 2 China State key Laboratory of Robotics, Shenyang Institute of Automation, Chinese Academy of Sciences, Shenyang 110016, China; qinshujia@sia.cn; 3 Mechanical and Biomedical Engineering Department, City University of Hong Kong, Tat Chee Avenue, Kowloon, Hong Kong, China; xintang2-c@my.cityu.edu.hk

**Keywords:** single pixel camera, motion estimation, video sampling, compressive sensing

## Abstract

Compressive sensing (CS) theory has opened up new paths for the development of signal processing applications. Based on this theory, a novel single pixel camera architecture has been introduced to overcome the current limitations and challenges of traditional focal plane arrays. However, video quality based on this method is limited by existing acquisition and recovery methods, and the method also suffers from being time-consuming. In this paper, a multi-frame motion estimation algorithm is proposed in CS video to enhance the video quality. The proposed algorithm uses multiple frames to implement motion estimation. Experimental results show that using multi-frame motion estimation can improve the quality of recovered videos. To further reduce the motion estimation time, a block match algorithm is used to process motion estimation. Experiments demonstrate that using the block match algorithm can reduce motion estimation time by 30%.

## 1. Introduction

Compressive sensing is a novel sampling theory. According to this theory, a small group of non-adaptive linear projections of a compressible signal contain enough information for image reconstruction and processing. Based on the compressive sensing theory, a new compressive imaging camera has been proposed [1]. The camera can recover an image with a single detection element while sampling the image fewer times than the number of pixels. Therefore, this camera is called as single pixel camera. This new hardware system mainly employs one photo-sensing device and a digital micro-mirror device (DMD). By consideration of a new theory of CS that combines sampling and compression procedures, this sensing method can recover a static image [2].

With the great success of single static image recovery, researchers begin to employ CS in video recovery. One direct way to implement CS video is to reconstruct a series of images by applying the recovery method to each frame independently. However, these methods do not utilize the motion information between each frame and have poor performance in terms of recovered video quality. Therefore, some research groups have considered the relationship between an image’s frames in a video, and motion estimation. The motion estimation parameters are imposed as the constraints in the image recovery procedure. The result achieves significantly higher quality than straightforward reconstruction that applies a still-image reconstruction independently. Some researchers use block matching (BM) to estimate motion between a pair of frames, and then combine motion estimation algorithms with image compression techniques [3,4,5]. Noor identified static and dynamic regions of arbitrary shapes for each frame [6], and the only dynamic moving regions are used for motion estimation. However, the mentioned methods treat scenes as a sequence of static frames as opposed to a continuously changing scene.

Another method is based on modeling specific video sequence evolution as a linear dynamical system (LDS) that requires a considerable number of samples for reconstruction [7]. However, the method is restricted to videos possessing an LDS representation, which is possible for only a few specific sequences. Sankaranarayanan *et al.* proposed a novel CS multi-scale video (CS-MUVI) sensing and recovery framework which presents a novel sensing matrix to achieve multi-scale sensing [8]. Besides, CS-MUVI extracts inter-frame motion information to improve the quality of reconstructed videos. However, CS-MUVI has two main drawbacks: (i) the motion estimation procedure needs a long time to employ an optical flow method; (ii) the inter-frame motion information is fully utilized.

In order to address these problems, an improved video CS scheme is proposed. In the proposed scheme, we utilize multi-frame motion estimation to improve the video quality. In addition, a block matching algorithm is employed in the motion estimation procedure to reduce the recovery time. In the paper, we first introduce some background knowledge for compressive sensing and single pixel cameras. Secondly, we introduce the whole structure and process for recovering video. The block matching algorithm and the multi-frame motion estimation will be discussed in detail. Finally, experimental results will be shown to verify the performance of our proposed scheme.

## 2. Compressive Sensing

Compressive sensing theory is a novel developed way to compress data during an acquisition process, and to recover the original signal with less sampling data [9,10,11,12,13,14]. Suppose there is a real signal x, x in ${\mathbb{R}}^{N}$ can be represented in terms of a basis of N×1 vectors ${\left\{\psi_{i}\right\}}_{i=1}^{N}$, we can express the signal x as:

(1) $$x=\sum_{i}^{N}\theta_{i}\psi_{i}$$

On account of ${\left\{\psi_{i}\right\}}_{i=1}^{N}$ is orthogonal basis, so:

(2) $$\theta_{i}=\langle x,\psi_{i}\rangle =\psi_{i}^{T}x$$

or represented in matrix form:

(3) x=Ψθ

Here, x is the representation of a signal in time domain, θ is the representation of a signal in Ψ domain. If only K elements of the θ in Equation (3) are nonzero and N−K are zero, we will call x in Ψ domain is K sparse. A linear measurement process can be considered as $y_{i}=\langle x,\Phi_{i}\rangle$ that computes M<N inner products between x and a collection of vectors ${\left\{\Phi_{j}\right\}}_{j=1}^{N}$. The measurement $y_{j}$ is stacked into the M×1 vector y, the measurement vectors $\Phi_{j}^{T}$ is stacked as rows into an M×N matrix Φ, so the expression is shown as:

(4) y=Φx=ΦΨθ

In order to recover the signal *x*, one needs to solve the following optimization problem:

(5) $$\hat{x}=\arg\underset{x\in {\mathbb{R}}^{N}}{\min}{\left\|\Psi^{T}x\right\|}_{0}\;s.t.\;y=\Phi x$$

Since the number of unknowns is greater than the number of equations, the equations have an infinite number of solutions, and it is need to find the optimal solution. According to compressive sensing theory, when ΦΨ satisfies the Restricted Isometry Property (RIP), the equation is able to accurately recover the sparse signal. For compressive sensing methods, this mainly includes two parts: (1) measurement matrix; and (2) recovering algorithm.

### 2.1. Measurement Matrix

It is preferred that the measurement matrix be sparse. The advantages of sparse measurement matrices include low computational complexity in both encoding and recovery, easy incremental updates to signals, and low storage requirements, *etc.* [15]. Some measurement matrices are random permutation matrices, such as Gaussian random variables as non-zero entries [16]. However, random matrices with high probability are incoherent with many basis, therefore, some measurement matrices are originated from special transforms such as Fourier, Cosine, or Walsh Hadamard transforms that are able to handle the fast computation of matrix-vector multiplication. In this paper, Walsh Hadamard transforms are used for measurement matrix generation.

### 2.2. Signal Reconstruction Algorithm

The signal reconstruction algorithm is one of the most important steps for compressed sensing and affects the reconstruction quality. The main calculation methods are $l_{1}$ minimization [17], greedy algorithm such as OMP [18] and total variation (TV) minimization scheme based on augmented Lagrangian and alternating direction algorithms (TVAL3) [19].

The TVAL3 algorithm is an efficient algorithm to recover an image for single pixel cameras. Therefore, the method is used in this paper for recovering images and it is described as follows:

(1) The TV model can be described by Equation (6):
  (6) $$\underset{w_{i},u}{\min}\sum_{i}\left\|w_{i}\right\|\;s.t.\;Au=b\;\mathrm{and}\;D_{i}u=w_{i}$$
  where $D_{i}$ is the discrete gradient of u at pixel i.
(2) The corresponding augmented Lagrangian problem is described by Equation (7):
  (7) $$\underset{w_{i},u}{\min}\sum_{i}(\left\|w_{i}\right\|-\alpha_{i}^{T}(D_{i}u-w_{i})+\frac{\beta_{i}}{2}{\left\|D_{i}u-w_{i}\right\|}^{2})-\gamma^{T}(Au-b)+\;\frac{\mu }{2}{\left\|Au-b\right\|}^{2}$$
(3) An alternating minimization scheme is applied to solving Equation (6). For a fixed u, the minimizing $w_{i}$ for all *i* can be obtained via Equation (8):
  (8) $$w_{i}=\max\left\{\left\|D_{i}u-\frac{\alpha_{i}}{\beta_{i}}\right\|-\frac{1}{\beta_{i}},0\right\}\frac{D_{i}u-\frac{\alpha_{i}}{\beta_{i}}}{\left\|D_{i}u-\frac{\alpha_{i}}{\beta_{i}}\right\|}$$
  where $\alpha_{i}$ and γ can be calculated as follows:
  (9) $$\alpha_{i}\leftarrow \alpha_{i}-\beta_{i}(D_{i}\hat{u}-{\hat{w}}_{i})$$
  (10) $$\gamma \leftarrow \gamma -\mu (A\hat{u}-b)$$
  here, μ is primary penalty parameter and $\beta_{i}$ is secondary penalty parameter. For fixed $w_{i}$, u is taken one steepest descent step with the step length computed by a back-tracking on-monotone line search scheme [20] starting from a Barzilai-Borwein (BB) step length [21]:
  (11) $$u_{k+1}=u_{k}-\varepsilon_{k}d_{k}$$

Here, $d_{k}=\sum_{i}(\beta_{i}D_{i}^{T}(-D_{i}u_{k}-w_{i,k+1})-D_{i}^{T}\alpha_{i})+\mu A^{T}(Au_{k}-b)-A^{T}\gamma$ and $\varepsilon_{k}=\frac{l_{k}^{T}y_{k}}{y_{k}^{T}y_{k}}$ where $l_{k}=u_{k}-u_{k-1}$ and $y_{k}=d_{k}(u_{k})-d_{k}(u_{k-1})$.

## 3. Compressed Video Sampling Perception

Almost every single pixel video camera scheme relies on a static scene model, which assumes that the scenes to be acquired are static. However, scenes are changing over time in the real world. This causes the problem that the recovered frames of video may have aliasing artifacts because video sampling cannot be finished in a very short time. In existing video compression schemes, motion estimation is used to reduce the storage cost. Inspired by the video coding schemes, many compressive sensing video recovery methods exploit motion estimation to enhance the recovered video quality. However, video frames are required while the motion estimates are the prerequisite for recovering video frames. Hence, a novel video sampling method is needed to solve these problems.

### 3.1. Single Pixel Video Camera Model

The principle of a single pixel video camera is similar to that of a single pixel camera that controls the optical path using a digital micro-mirror device (DMD) to achieve linear measurements of an image. In order to record a video, a single pixel video camera needs to continuously sample a scene because a video consists of a series of frames. Suppose that at sample instant t, $\phi_{t}\in {\mathbb{R}}^{N\times 1}$ is the measurement vector, $x_{t}\in {\mathbb{R}}^{N\times 1}$ is the scene and $e_{t}\in \mathbb{R}$ is the measurement noise, the measurement process can be modeled as:

(12) $$y_{t}=\langle \phi_{t},x_{t}\rangle +e_{t}$$

Suppose 1≤t≤T and *T* is the total number of measurements, $y_{1:W}$ can be used to represent W successive measurements wher W≤T:

(13) $$y_{1:W}=\left[\begin{array}{c} y_{1}\\ y_{2}\\ \vdots \\ y_{W} \end{array}\right]=\left[\begin{array}{c} \langle \phi_{1},x_{1}\rangle +e_{1}\\ \langle \phi_{1},x_{2}\rangle +e_{2}\\ \vdots \\ \langle \phi_{W},x_{W}\rangle +e_{W} \end{array}\right]$$

### 3.2. Errors in the Static Scene Model

In reality scenes are varying over time, thus $x_{t}$ is time varying. Therefore, the frames recovered from $y_{1:W}$ may be distorted. We can rewrite $x_{t}$ as:

(14) $$x_{t}=b+\Delta x_{t}$$

where b is the static component of the scene, and $\Delta x_{t}$ represents the dynamic component which can also be considered as the error caused by violating static scene assumption at instant t. In this situation, $y_{1:W}$ can be represented as follows:

(15) $$y_{1:W}=\left[\begin{array}{c} y_{1}\\ y_{2}\\ \vdots \\ y_{W} \end{array}\right]=\left[\begin{array}{c} \langle \phi_{1},b\rangle +\langle \phi_{1},\Delta x_{1}\rangle +e_{1}\\ \langle \phi_{1},b\rangle +\langle \phi_{1},\Delta x_{1}\rangle +e_{1}\\ \vdots \\ \langle \phi_{W},b\rangle +\langle \phi_{W},\Delta x_{W}\rangle +e_{W} \end{array}\right]$$

Let $z_{t}=\langle \phi_{t},\Delta x_{t}\rangle$, the above equation can be rewritten as:

(16) $$y_{1:W}=\Phi b+z_{1:W}+e_{1:W}$$

From Equation (16) we can see that when the scenes are varying ($x_{t}\neq b$), an error $z_{1:W}$ caused by the violation of the static scene assumption is produced.

### 3.3. CS-MUVI Scheme

Many video sampling schemes based on compressive sensing do not take into account the errors caused by violating the static scene assumption. Sankaranarayanan *et al.* [8] analyzed these errors and proposed the compressive sensing multi-scale video (CS-MUVI) sensing and recovery framework. Based on the analysis of dynamic scene, they developed a novel video sampling technology to overcome the static scene assumption. A novel sensing matrix referred to as dual-scale sensing (DSS) matrix that realizes the computation of a low-resolution frame is designed and implemented. To improve the recovered video quality, they exploit the optical flows from low-resolution frames, which will be imposed as constraints in the final recovery procedure.

**Figure 1:**
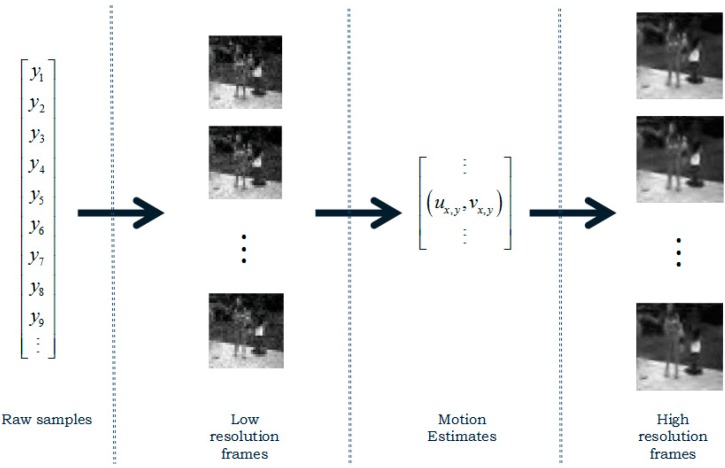
CS-MUVI Procedure.

CS-MUVI employs least square (LS) method to compute and recover the low-resolution frame. Therefore, W successive measurements can reconstruct $x\in {\mathbb{R}}^{W}$ which can be seen as an image of $\sqrt{W}\times \sqrt{W}$ resolution. Using the LS method, it can recover low-resolution frames from a few measurements. Fewer measurements need less measurement time, thus the dynamic scene can be perceived as a static scene, which reduces the error caused by violating the static scene assumption. In the dynamic scene model, this method can acquire robust low-resolution frames. According to the CS-MUVI framework, three major steps are included as shown in Figure 1. Firstly, LS method is used to recover the low-resolution frames. Secondly, optical flow computation method is used to compute the motion estimation between the recovered low-resolution frames. Finally, the motion estimation constraints are imposed as constraints in the final recover procedure that recover the high-resolution frames.

Low-resolution frames can be obtained by down-sampling high-resolution frames. Let $b\in {\mathbb{R}}^{N_{L}}$ be the static component of a low-resolution frame and suppose $N_{L}=n_{L}\times n_{L}$, $N_{L}<N$. Meanwhile, we define $U\in {\mathbb{R}}^{N\times N_{L}}$ as up-sampling operator and $D\in {\mathbb{R}}^{N_{L}\times N}$ as down-sampling operator. According to Equation (13), we have:

(17) $$\begin{array}{ll} y_{1:W} & =\Phi b+z_{1:W}+e_{1:W}\\ & =\Phi (Ub_{L}+b-Ub_{L})+z_{1:W}+e_{1:W}\\ & =\Phi Ub_{L}+\Phi (I-\mathrm{UD})b+z_{1:W}+e_{1:W} \end{array}$$

where $b_{L}=Db$. From Equation (17), the measurements of low-resolution static scene has three sources of error: (1) the error Φ(I-UD)b caused by down-sampling; (2) the error $z_{1:W}$ caused by violating static scene model; (3) the measurement error $e_{1:W}$.

As LS method is used to compute the low-resolution frames, the number of measurements to recover must be larger than $N_{L}$, *i.e.*, $W\geq N_{L}$. Assume ΦU is of dimension $W\times N_{L}$ with $W\geq N_{L}$, we can estimate the low-resolution static scene $b_{L}$ from Equation (14) using LS method:,

(18) $$\begin{array}{l} \hat{b}={\left(\Phi U\right)}^{†}y_{1:W}\\ \;=b_{L}+{\left(\Phi U\right)}^{†}(\Phi (I-\mathrm{UD})b+z_{1:W}+e_{1:W}) \end{array}$$

where † denotes the pseudo inverse operation. From Equation (18) we observe that ${\hat{b}}_{L}$ has three errors which are Φ(I-UD)b, $z_{1:W}$ and $e_{1:W}$. W determines Φ(I-UD)b and $z_{1:W}$. If W increases, the error Φ(I-UD)b will decrease. However, a larger W requires more time to finish the sensing process, thus making the error $z_{1:W}$ bigger. Therefore, W must be selected properly in light of different situations. Note that ${\left(\Phi U\right)}^{†}$ may potentially amplify three errors [8].

#### 3.3.1. Sensing Matrix

CS-MUVI comes up with a novel class of sensing matrices, referred to as dual-scale sensing (DSS) matrices. DSS matrices are able to improve the recovery performance and reduce the error enhancement when computing a low-resolution frame. These matrices can satisfy RIP and remain well-conditioned even if they are multiplied by a given up-sampling operator, *i.e.*, ${\left(\Phi U\right)}^{†}$. These properties make DSS matrices a suitable choice in the process of video sampling.

In the single pixel camera architecture, the micromirrors only determine two states, thus each entry of DSS matrices is binary-valued. For convenience and better performance, we let ΦU=H, where H is a W×W Hadamard matrix. In order to satisfy all the requirements mentioned above, Φ is created as follows:

(19) Φ=HD+F

where $F\in {\mathbb{R}}^{W\times N}$ is an auxiliary matrix that meets the following requirements: (1) the elements of Φ is either 1 or −1; (2) Φ can satisfy RIP; (3) FU=0. Sankaranarayanan *et al.* proposes that choosing random entries in F by using random patterns of high spatial frequency achieves excellent performance.

#### 3.3.2. Motion Estimation

**Figure 2:**
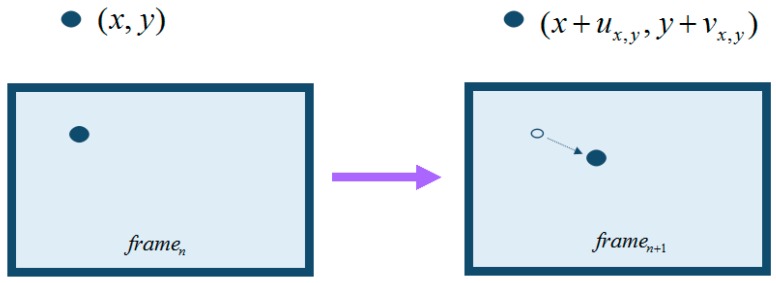
Optical flow.

After obtaining the low-resolution frames of a video, we can use them to perfrom motion estimation. CS-MUVI employs optical flow computation to obtain motion estimation. Optical flows can be considered as the motions of pixels caused by objects’ motion between two consecutive frames as shown in Figure 2. The study of optical flow comes from Horn, Chunck, Lucas and Kanade in 1980s [22].

Suppose we have obtained two successive low-resolution frames, named as ${\hat{b}}_{L}^{i}$ and ${\hat{b}}_{L}^{j}$. By up-sampling them we have full spatial resolution frames ${\hat{b}}^{i}=U{\hat{b}}_{L}^{i}$ and ${\hat{b}}^{j}=U{\hat{b}}_{L}^{j}$. Then the optical flows between ${\hat{b}}^{i}$ and ${\hat{b}}^{j}$ are computed and we have the following constraint:

(20) $${\hat{b}}^{i}(x,y)={\hat{b}}^{j}(x+u_{x,y},y+v_{x,y})$$

where ${\hat{b}}^{i}(x,y)$ denotes the pixel of ${\hat{b}}^{i}$ at position (x,y). $u_{x,y}$ is the pixel’s velocity in x direction and $v_{x,y}$ is the velocity in y direction. $u_{x,y}$ and $v_{x,y}$ can only represent velocity and cannot guarantee occlusion. To prevent occlusion, we need to compute the forward and backward optical flows to ensure the consistency between them.

Using optical flow motion estimation, CS-MUVI can effectively reduce the noise and artifacts caused by violating static scene assumption. However, this method is very time-consuming. In some circumstances, the time used to compute optical flows accounts for nearly half of the total time. Hence, we have to wait for a long time before the high-resolution frames are recovered.

#### 3.3.3. Block Match Algorithm

The block match algorithm is one of the most popular methods used to compute motion estimation, especially in the field of video coding. The block match algorithm acquires motion estimations of pixel blocks between two consecutive frames by computing the displacements [23]. It is a robust and efficient motion estimation algorithm due to its simple implementation and low computation cost. In this paper, we propose a block match algorithm to replace optical flow method in the motion estimation, so that the time can be reduced.

The block match algorithm divides a frame into many small non-overlapping pixel blocks by grouping the pixels in a small window. For example, the number of pixel blocks with size of p×q in a h×w frame is (h/p)×(w/q). Normally the value of p and q is 4 or 8. For each pixel block, the block match algorithm searches for the most similar pixel block in the neighboring frame. If such a pixel block is found, we can acquire the motion estimates by computing the block displacement. We define $b^{m}(x\times p+i,y\times q+j)$ as the pixel (i,j) in the block (x+1,y+1) of frame *m*. For simplicity, we use $b_{x,y}^{m}(i,j)$ to denote $b_{x,y}^{m}(x\times p+i,y\times q+j)$ below.

The simplest block match algorithm is the Full Search algorithm which means searching for the target block in the entire neighboring frame. Full search algorithms can yield the most accurate results but the time cost is also the highest. How to design a robust and fast block match algorithm has become a big challenge and many researchers are working on it and have put forward many fast block match algorithms. Nonetheless, these fast algorithms aim at high definition videos of which the resolutions are much higher than that of videos recovered by compressive sensing. Hence we implement a new block match algorithm that aims at a compressive sensing video environment. The Algorithm 1 details are given below:   **Algorithm 1:**  *Initialize*
sad←inf, dif←0  *For* each block $b_{x,y}^{n}$ in frame n
*Do*
$$x^{\prime }\leftarrow x,y^{\prime }\leftarrow y;$$  *While* stop criteria unsatisfied *Do*  $B\leftarrow \{(u,v):\left|u-x^{\prime }\right|+\left|v-y^{\prime }\right|=1\}$   *For* each (u,v)∈B
*Do*  $dif=\sum_{i=1}^{p}\sum_{j=1}^{q}\left|b_{x,y}^{m}(i,j)-b_{u,v}^{n}(i,j)\right|,$  *if*
dif<sad
*then*  sad←dif;  R(x,y)←(u−x,v−y);  *End if*    *End Do*    $x^{\prime }\leftarrow u$, $y^{\prime }\leftarrow v$;  *End Do*  *End Do**Output*
R(x,y) of each block $b_{x,y}^{n}$.

Compared to the block match algorithm, optical flow method has the advantage of higher accuracy. However, it needs to compute both forward and backward flows, so it can prevent occlusions. In addition, the optical flow method manipulates pixels rather than blocks, thus it requires higher computational complexity. On the contrary, the block match algorithm does not have occlusion problems, and its manipulated objects are blocks instead of pixels. Besides, the search task in each block can be executed independently in parallel. If a computer has enough operation cores, the time cost can be reduced significantly. Table 1 gives the detailed comparison between optical flow method and block match algorithm. It is worth noting that there exist other motion estimation methods. For example, Kuglin proposed a phase correlation algorithm that calculates relative translative offset between two similar images [24]. This method estimates the relative translative offset between two similar images and has been successfully applied in video. However, this method only considers the overall offset between two images, which is not suitable for local pixels offset. Therefore, we choose block-matching algorithm to compute the displacements of pixels between each frame. Much of the noise in the high-resolution frames is caused by the inaccuracies of the motion estimates caused by up-sampling low-resolution frames. Therefore, improving the motion estimation can potentially contribute to the final recovery step. The continuity of object movement may maintain across multiple frames. Inspired by Rubinstein and Liu, the authors of CS-MUVI envision significant performance improvements if multi-frame motion estimation is used [25].

In this paper, we apply multi-frame motion estimation to CS-MUVI and conduct several experiments to validate the performance. Suppose we have a certain low-resolution Frame *i* and associate it with Frame i + 1, Frame i + 2, …, Frame i + n respectively to form n pairs of frames. Assume n is 3, then we can obtain motion estimates $\left(u_{x,y}^{i+1},v_{x,y}^{i+1}\right)$, $\left(u_{x,y}^{i+2},v_{x,y}^{i+2}\right)$, $\left(u_{x,y}^{i+3},v_{x,y}^{i+3}\right)$ that is used to generate the following motion estimation constraints:

(21) $$\{\begin{array}{c} {\hat{b}}^{i}(x,y)={\hat{b}}^{i+1}(x+u_{x,y}^{i+1},y+v_{x,y}^{i+1})\\ {\hat{b}}^{i}(x,y)={\hat{b}}^{i+2}(x+u_{x,y}^{i+2},y+v_{x,y}^{i+2})\\ {\hat{b}}^{i}(x,y)={\hat{b}}^{i+3}(x+u_{x,y}^{i+3},y+v_{x,y}^{i+3}) \end{array}$$

#### 3.3.4. Recovery of High-Resolution Frames

After computing the motion estimation, the high-resolution frames are ready to be recovered. One notion needed to be specified before the final recovery step is the rate of frames to be recovered, *i.e.*, frames interval ΔW. ΔW must ensure sub-pixel ΔW motion between consecutive frames. In terms of a rapidly moving scene, a smaller ΔW can achieve better results. As for a slowly moving scene, a bigger ΔW can reduce the computation cost. It can be chosen according to the low-resolution frames.

Suppose that ΔW is chosen properly and $\Delta W=N_{L}$, the high-resolution frames can be recovered by solving the following convex optimization problem:

(22) $$\{\begin{array}{c} \underset{x}{\min}\mathrm{TV}(x)\\ \begin{array}{l} s.t.\;{\left\|\langle \phi_{t},x_{I(t)}\rangle -y_{t}\right\|}_{2}\leq \varepsilon_{1}\\ \;\{\left\|\begin{array}{c} \vdots \\ x_{i}(x,y)-x_{i+1}(x+u_{x,y}^{i+1},y+v_{x,y}^{i+1})\\ x_{i}(x,y)-x_{i+2}(x+u_{x,y}^{i+2},y+v_{x,y}^{i+2})\\ x_{i}(x,y)-x_{i+3}(x+u_{x,y}^{i+3},y+v_{x,y}^{i+3})\\ \vdots \end{array}\right\|\leq \varepsilon_{2} \end{array} \end{array}$$

where TV denotes the total variation operator and I(t) maps the sample index t to associated frame index *k*. $\varepsilon_{1}$ is the indicative of the measurement noise levels caused by photon-noise, thermal and read noise. $\varepsilon_{2}$ is the inaccuracies of brightness constancy which can be set by detecting the floating range of brightness constancy.

## 4. Experiments

In order to validate the performance of our proposed method, we carried out several experiments and compared the results in terms of peak signal-to-noise ratio (PSNR) and time cost. The PSNR is commonly used to measure the quality of recovered image. A higher PSNR generally indicates that the reconstruction is of higher quality and it is defined as:

(23) $$PSNR=10\log_{10}\frac{{\left(2^{n}-1\right)}^{2}}{MSE}$$

where *MSE* is the mean squared error and *n* it the number of bit.

The sample data is a video file with six frames, and we compared the recovered images with the original images in the sample data. The images are recovered in a high performance PC (Intel i7 3720QM 2.6 GHz CPU with 8 GB memory) using Matlab 2012a under Windows 7.

### 4.1. Experimental Setup of the Single Pixie Video Camera

**Figure 3:**
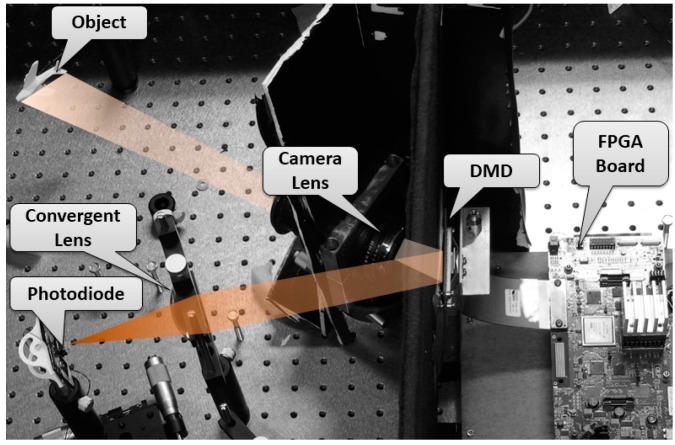
Experimental setup of the single pixel camera system.

The experimental setup is shown in Figure 3. It mainly consists of a DMD, one photo detector, some lenses, and the control circuit.

#### 4.1.1. Components of the SYSTEM

A DMD (DLP^®^ Discovery™ 4100, Texas Instruments, Inc., Dallas, TX, USA) is used to generate the measurement matrix. Each DMD micromirror can be controlled independently to two different positions, so different patterns on the digital micromirror device are equivalent to different measurement matrices. The mirror resolution is 1024 × 768, Global Reset Max FPS is 22,614 and Phased Reset Max FPS is 32,552. Wavelength range is about 300–2800 nm. One camera lens (SIGMA camera lens model, focal length range is 28–138 mm and F3.8–5.6) and two convex lens (Focal length is 70 mm and size diameter is 60 mm) are used. The camera lens is used as lens system to make real object project on the DMD. And its focal length range is 28–138 mm so that different objects can be projected on the DMD. A convex lens are used to collect the reflection light of the DMD to a photodiode. The photodiode (OPT101, Burr-Brown Corporation, Tucson, AZ, USA) is employed in the system. The chip is a monolithic photodiode with on-chip transimpedance amplifier. Output voltage increases linearly with light intensity. The detectable wavelength of the chip is from 500 nm to 1000 nm. The DMD data access is organized in sequences of XGA frames, which is controlled by FPGA. A series of patterns need be displayed at high rates and all patterns in a sequence have the same bit-depth and different sequences may be defined and loaded at the same time. A high performance 24-bit Σ-Δ analog-to-digital converter (ADC) chip (AD7760, Analog Devices, Inc., Norwood, MA, USA) for the signal conversion.

#### 4.1.2. The Workflow of the Single-Pixel Camera

**Figure 4:**
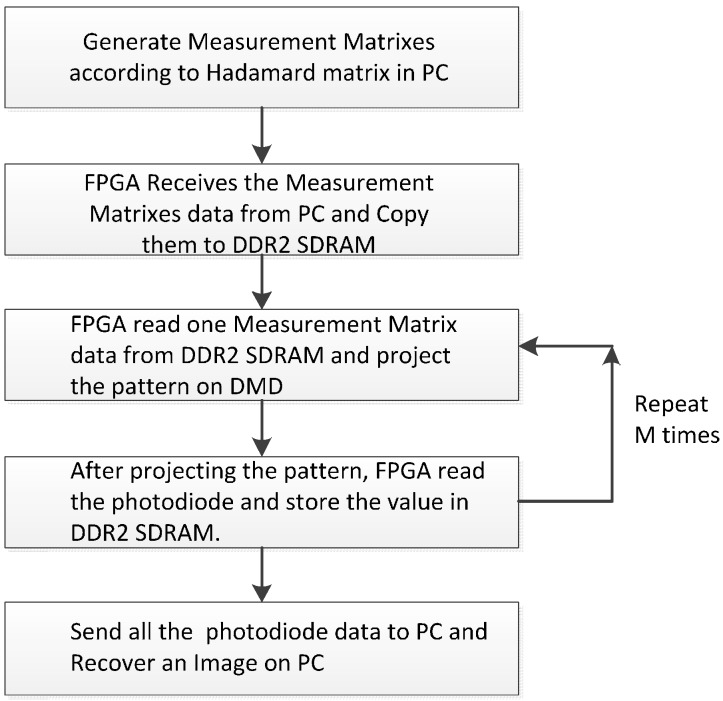
The workflow of single-pixel camera.

The workflow of the single-pixel camera is shown in Figure 4. Firstly, the Measurement Matrix is generated and the pattern data transported to the DDR2 SDRAM. Secondly, the pattern data is read from the DDR2 SDRAM to project on the DMD and the photodiode’s value read to memorize to the DDR2 SDRAM. The process will repeat M times (M is the number of measurements). Finally, the A/D sample values will be sent to PC from DDR SDRAM2 and the image will be recovered.

#### 4.1.3. Parallel Control System Based on FPGA

**Figure 5:**
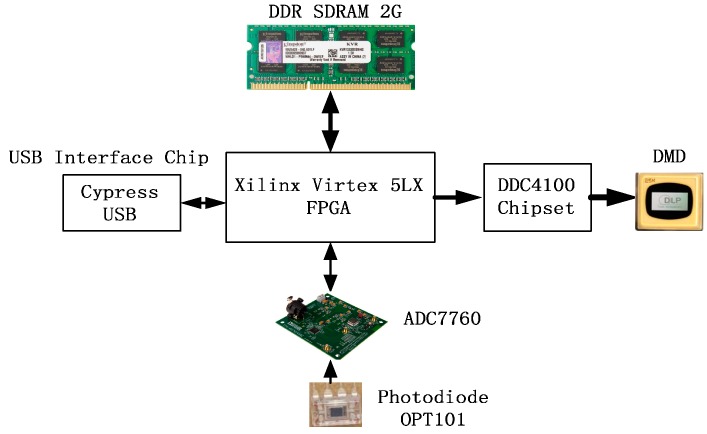
The diagram of single pixel camera.

FPGA is used to solve real-time processing of Single-Pixel imaging which is challenging because of the inherent instruction cycle delay in transporting data on a DMD. A FPGA board (Virtex-5, Xilinx Inc., San Jose, CA, USA) is used to control the DMD in high speed as shown in the Figure 5.

The hardware includes a Virtex-5 FPGA linking the on-board DDR2 SDRAM pattern sequence memory. The SDRAM on-board memory stores the pattern sequences that are pre-loaded for subsequent high-speed display. The Virtex 5 FPGA connects with DDC4100 chip by a parallel interface and transfers pattern data to it directly. At the same time, DDC4100 controls the mirrors to turn for generating measurement matrices according to the pattern data. The board includes an USB controller that enables the PC connection for data transfer.

**Figure 6:**
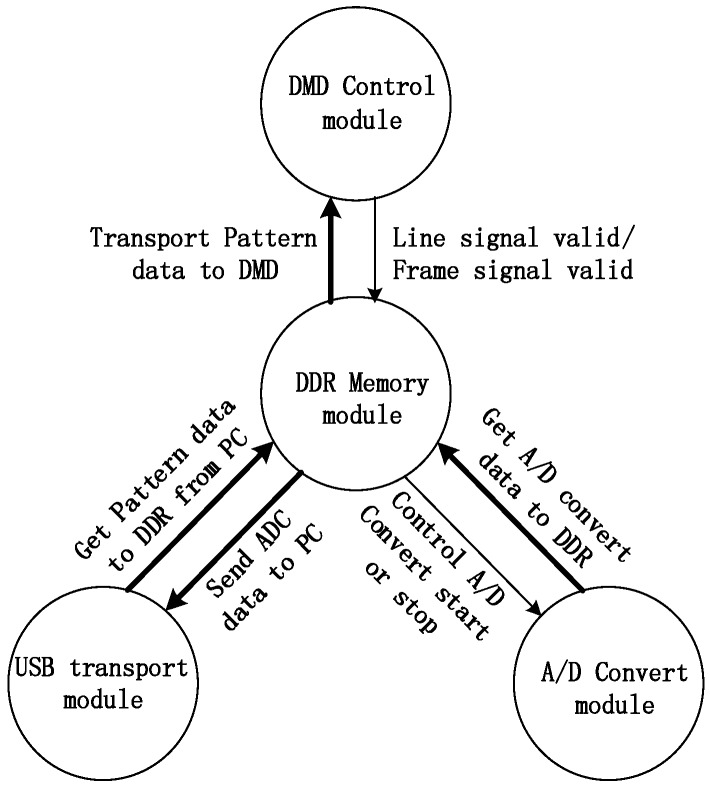
The relation schema of the main modules.

After transmitting one pattern, the Virtex-5 FPGA needs to sample analog the voltage of photodiodes through an A/D converter. Then the sample result is sent to a PC through the USB interface. At the end of process, the picture will be recovered on a PC according to the sample values. Therefore, we design the system consists of four modules as shown in Figure 6, they includes DMD control module, DDR2 SDRAM memory module (SO-DIMM-connected 2G-Bit DDR2SDRAM), A/D converter module and USB transport module.

### 4.2. Multi-Frame Motion Estimation

**Figure 7:**
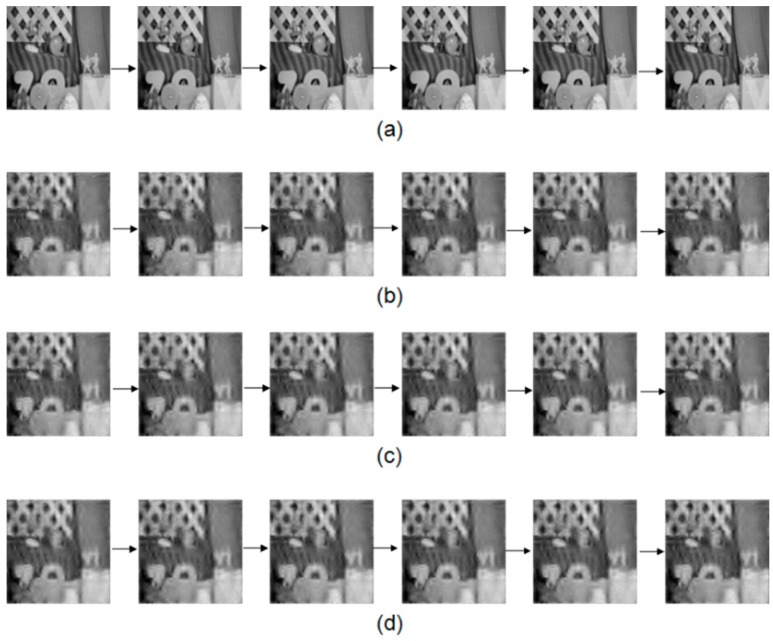
(**a**) The images from the original sample video; (**b**) The recovered video using only two frame motion estimation (as used in the CS-MUVI); (**c**,**d**) are recovered video using three and four frame motion estimation, respectively.

**Figure 8:**
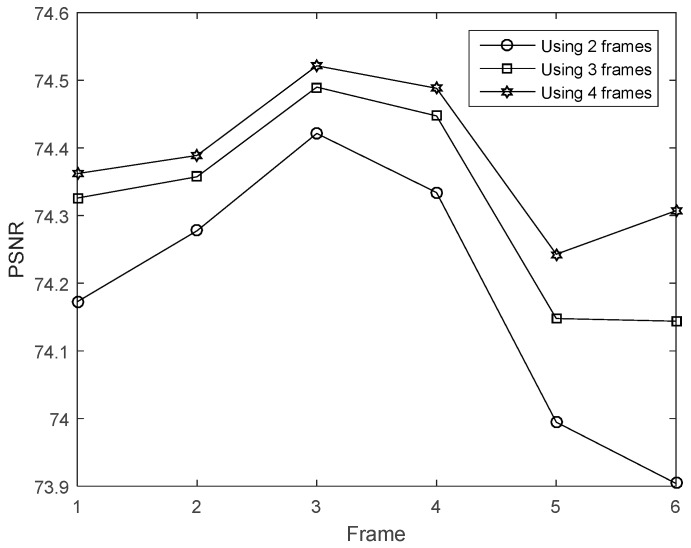
PSNR of the image recovered using 2, 3 and 4 frames for multi-frame motion estimation.

**Figure 9:**
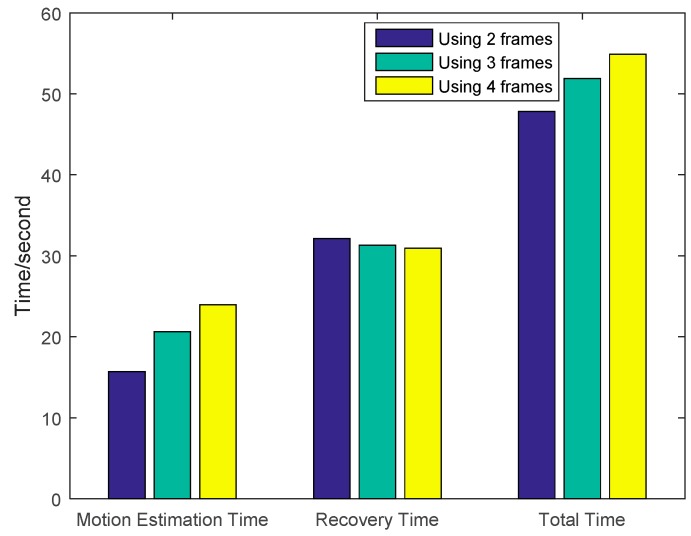
Time cost of the images recovered using 2, 3 and 4 frames for multi-frame motion estimation.

The first experiment is to validate the performance of improved method that uses multi-frame motion estimation. The motion estimation is achieved via an optical flow computation method. The parameters are set according to [8]. The low-resolution frame’s size is 32 × 32 and the high resolution frame’s size is 128 × 128. In this experiment, we compared the image quality by using different number of frames (2, 3 and 4 frames) for the motion estimation. We can see that the quality of Figure 7c,d is better than that of Figure 7b. The images in Figure 7d also looks closer to Figure 7a. Besides, the PSNR using the multi-frame motion estimation is shown in Figure 8. Results indicates that four frame motion estimation achieves the best performance in terms of PSNR ranging from 74.2 to 74.5.The time costs using different recovery methods are compared in Figure 9. Using 4 frames need a little more time comparing with 2 frames and 3 frames.

### 4.3. Block Match Motion Estimation

**Figure 10:**
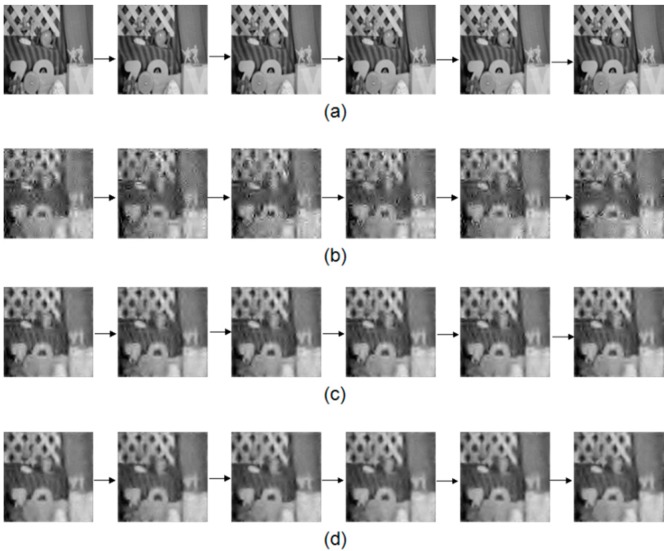
(**a**) The images from the original sample video; (**b**) The video recovered by the traditional compressive sensing algorithm; (**c**) The video recovered by CS-MUVI; and (**d**) the video recovered using our proposed block match motion estimation.

This experiment demonstrates the performance of the block match motion estimation. We used CS-MUVI and traditional scheme to recover the videos for comparison as shown in Figure 10.

**Figure 11:**
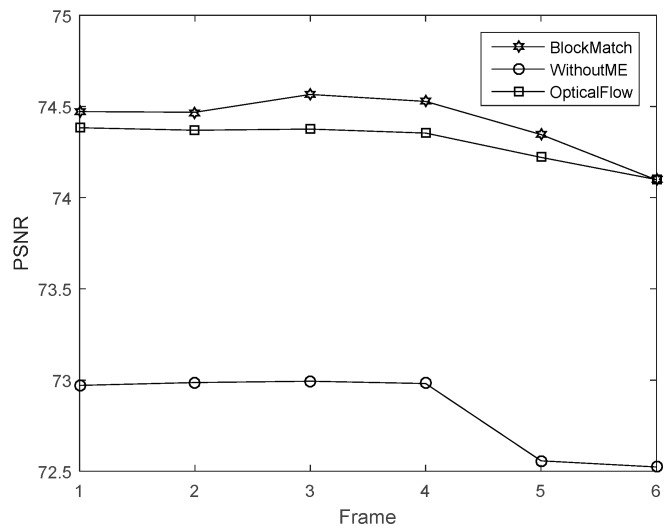
PSNR of the images reovered by our block matching scheme, the optical flow and without motion estimation (WithoutME).

The sample videos are the same. The optical flow computation parameters are set according to [8]. In the block match algorithm, the block size is 4 × 4, and the maximum search range is 4. Figure 11 presents the corresponding PSNR. In this experiment, our proposed scheme has the best performance.

**Figure 12:**
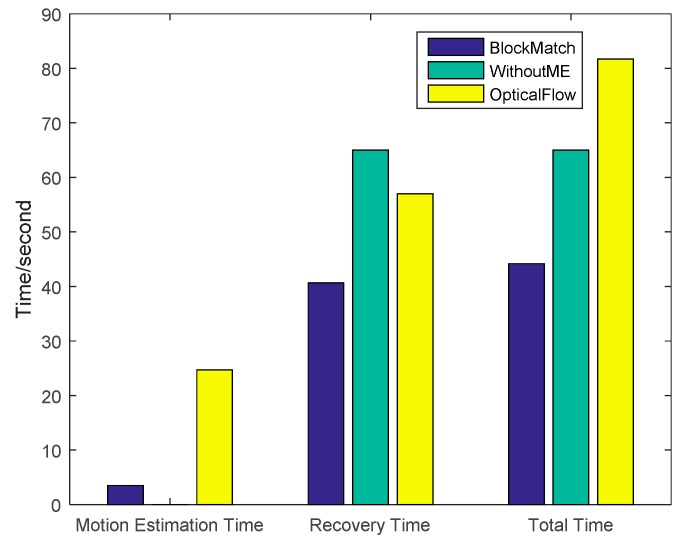
Time cost of the the images reovered by our block matching Scheme, the optical flow and without motion estimation (WithoutME).

The time costs using different recovery methods are compared in Figure 12. The traditional method has the lowest time cost while CS-MUVI has the highest. The time cost of our proposed method is 15.6% larger than traditional method and 32.7% less than CS-MUVI.

**Figure 13:**
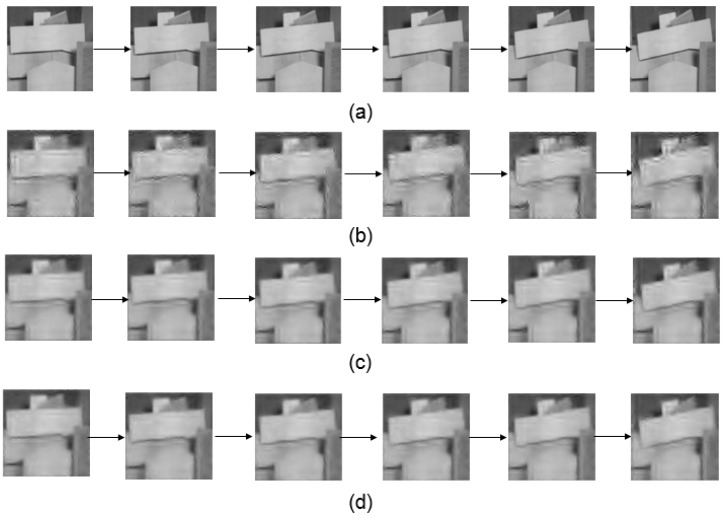
(**a**) The images from the second sample video; (**b**) The video recovered by traditional compressive sensing algorithm; (**c**) The video is recovered by CS-MUVI; (**d**) The video is recovered using our proposed block match motion estimation.

**Figure 14:**
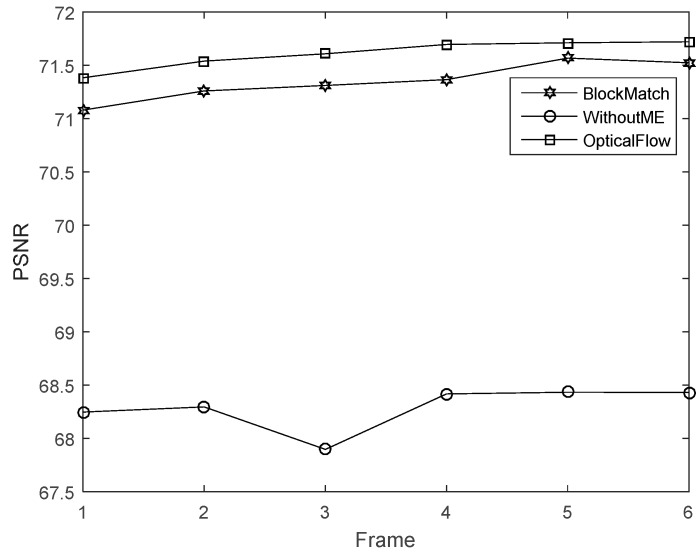
PSNR of the images reovered by our block matching scheme, the optical flow and without motion estimation (WithoutME).

**Figure 15:**
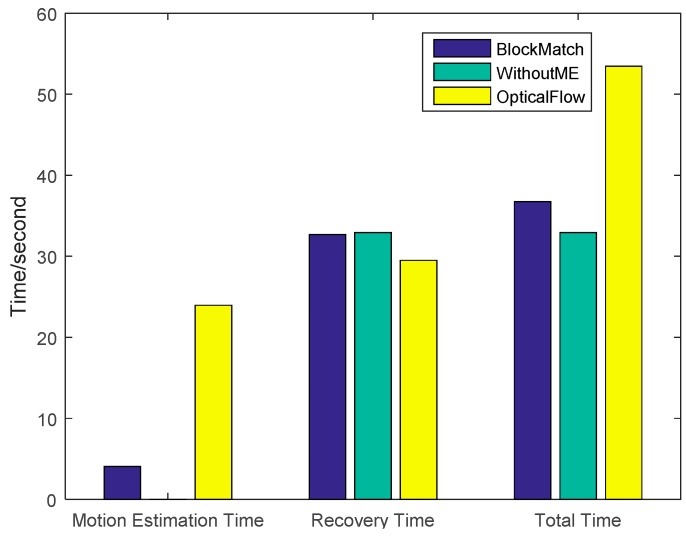
Time cost of the images recovered by our block matching scheme, the optical flow and without motion estimation (WithoutME).

To further demonstrate that our algorithm, we used another sample video for further testing and the results are shown in Figure 13. The recovery accuracy is shown in Figure 14 and the time cost is shown in Figure 15. From the results we can see that, after using motion estimations, the recovered accuracy has improved with PSNR increasing from 68.3 to about 75.3. The CS-MUVI which uses optical flow algorithm has slightly higher accuracy than our approach. However, the total cost of our approach is about 38 s which is faster than the CS-MUVI method (~55 s).

### 4.4. Analysis of Experiments

From the multi-frame motion estimation experimental results, it is seen that by adding a number of frames to perform motion estimation one increase the description of the original videos, and thus improve the quality of recovered video. However, this improvement will increase the motion estimation time because the computation complexity is also increased. Nonetheless, the action does not necessarily increase the recovery time.

In the second and third experiment, we can see that our improved method and the optical flow estimation method can efficiently solve the problems caused by violating the static scene model. By replacing the optical flow method with our block match algorithm, the motion estimation time can be reduced and the recovery time may potentially decrease. Even though the block match algorithm uses a coarse precision search strategy, it may find local optimal solution in some cases, and it can accelerate recovery process. It is obvious that using multi-frame motion estimation can improve the recovered video quality. Besides, our proposed scheme that adopts block match algorithm is faster than the CS-MUVI method that adopts optical flow motion estimation.

## 5. Conclusions

In this paper, we have discussed a new scheme to acquire motion estimation for video reconstruction. Our proposed scheme adopts a block match algorithm to reduce the motion estimation time, which is different from CS-MUVI that uses optical flow methods. The block match algorithm is a simple and efficient method to acquire motion estimate between two consecutive frames. From the experiments, we can see that it can vastly reduce the motion estimation time. Besides, the multi-frame motion estimation algorithm is used to enhance the video quality. Instead of using two frames motion estimation, the proposed algorithm uses multiple frames to implement motion estimation. The experimental results show that this algorithm can improve the quality of the recovered videos and further reduce the motion blur.

## Figures and Tables

**Table 1:** Comparison between the optical flow methods and the block match algorithm.

	Optical Flow Method [22]	Block Match Algorithm [23]	Phase Correlation Algorithm [24]
Target	pixel velocity	block displacement	linear phase differences
Object	pixel	block	whole image
Method	iterative least square method	independent search	fourier Shift property
Cost	high	low	low
Accuracy	high	medium	low
Parallelization	low	high	low
